# Evidence of Maintenance Tagging in the Hippocampus for the Persistence of Long-Lasting Memory Storage

**DOI:** 10.1155/2015/603672

**Published:** 2015-08-25

**Authors:** Micol Tomaiuolo, Cynthia Katche, Haydee Viola, Jorge H. Medina

**Affiliations:** ^1^Instituto de Biología Celular y Neurociencias “Dr. Eduardo De Robertis”, Facultad de Medicina, Universidad de Buenos Aires, C1121ABG Buenos Aires, Argentina; ^2^Departamento de Fisiología, Biología Molecular y Celular Dr. Hector Maldonado, Facultad de Ciencias Exactas y Naturales, Universidad de Buenos Aires, C1428EGA Buenos Aires, Argentina; ^3^Departamento de Fisiología, Facultad de Medicina, Universidad de Buenos Aires, C1121ABG Buenos Aires, Argentina

## Abstract

The synaptic tagging and capture (STC) hypothesis provides a compelling explanation for synaptic specificity and facilitation of long-term potentiation. Its implication on long-term memory (LTM) formation led to postulate the behavioral tagging mechanism. Here we show that a maintenance tagging process may operate in the hippocampus late after acquisition for the persistence of long-lasting memory storage. The proposed maintenance tagging has several characteristics: (1) the tag is transient and time-dependent; (2) it sets in a late critical time window after an aversive training which induces a short-lasting LTM; (3) exposing rats to a novel environment specifically within this tag time window enables the consolidation to a long-lasting LTM; (4) a familiar environment exploration was not effective; (5) the effect of novelty on the promotion of memory persistence requires dopamine D1/D5 receptors and Arc expression in the dorsal hippocampus. The present results can be explained by a broader version of the behavioral tagging hypothesis and highlight the idea that the durability of a memory trace depends either on late tag mechanisms induced by a training session or on events experienced close in time to this tag.

## 1. Introduction

The synaptic tagging and capture (STC) hypothesis provided a strong framework to explain how to achieve synaptic specificity and persistence of electrophysiological-induced plasticity changes. It predicts that induction of long-term potentiation creates a synapse-specific “tagged state” that may capture diffusible plasticity-related proteins (PRPs) that are induced by other neural activity [1, 2]. The functional relevance of STC hypothesis and its implications for learning and long-term memory (LTM) formation led to postulate the behavioral tagging mechanism [3]. This process explains how weak trainings inducing a short-term memory and a “learning tag” can be established into LTM (lasting at least 24 h) if animals are exposed to a novel experience which provides the PRPs. Two important requirements for this process are the critical time window of efficacy, justified in part for the transient aspect of the tag and the novelty attribute of the associated experience [3, 4]. In the few past years, several research groups have worked on the behavioral tagging process demonstrating that it was observed in operant and Pavlovian aversive paradigms, in the formation of extinction and spatial object recognition memories and in other tasks based on spatial learning [2–14]. Moreover, a similar phenomenon was observed also in school children who had learnt about a tale or a drawing, suggesting the generality of the process in long-lasting memory formation [15]. Despite the plethora of information concerning the behavioral tagging in LTM formation, up till now there is no information about the existence of a maintenance tag underlying the process for long-lasting memory storage.

Given that a late BDNF- (brain-derived neurotrophic factor-) and protein synthesis-dependent phase of consolidation occurring around 12 h after strong inhibitory avoidance (IA) training in the dorsal hippocampus is required for memory persistence [16, 17], we predict that IA training generates a maintenance-specific tag late after training, which captures PRPs required for long-lasting memory storage. Then, we postulate that a weak IA training that generates a short-lasting LTM of a couple of days would just create a maintenance-specific tag while PRPs necessary for memory persistence would be provided by a close-in-time novel experience. As a consequence, the duration of the storage of the original IA memory would be much longer than expected, establishing a long-lasting LTM. Here, we present evidence of a late “tagged state” of the memory trace which is involved in the persistence of LTM storage through a maintenance tagging process.

## 2. Materials and Methods

### 2.1. Subjects

A total of 315 male Wistar rats from the vivarium of the Italian Hospital (Buenos Aires, Argentina) weighting 230–260 g were used. Animals were housed five to a cage and kept at a constant temperature of 22°C, with water and food* ad libitum*, under a 12 h light/dark cycle (lights on at 8:00 A.M.). Each animal was used only for one experiment. Experimental procedures followed the guidelines of the USA National Institutes of Health Guide for the Care and Use of Laboratory Animals and were approved by the Animal Care and Use Committees of the University Buenos Aires (CICUAL).

### 2.2. Surgery

Rats were bilaterally implanted under deep ketamine/xylazine anesthesia (100 and 5 mg/kg, resp.) with 22 g guide cannulae aimed at dorsal CA1 region of the hippocampus (AP −4.3 mm, LL ± 3.0 mm, DV 1.4 mm) (from Bregma). Coordinates were based on Paxinos and Watson (1997) [18]. Cannulae were fixed to the skull with dental acrylic. Obturators were then inserted into the cannulae to prevent blockage, with the same or less length of the cannulae. At the end of surgery, animals were injected with a single dose of meloxicam (0.2 mg/kg) as analgesic and gentamicin (2.5 mg/kg) as antibiotic. Behavioral procedures began 5–7 days after surgery.

### 2.3. Inhibitory Avoidance Training and Testing

**Figure 1:**
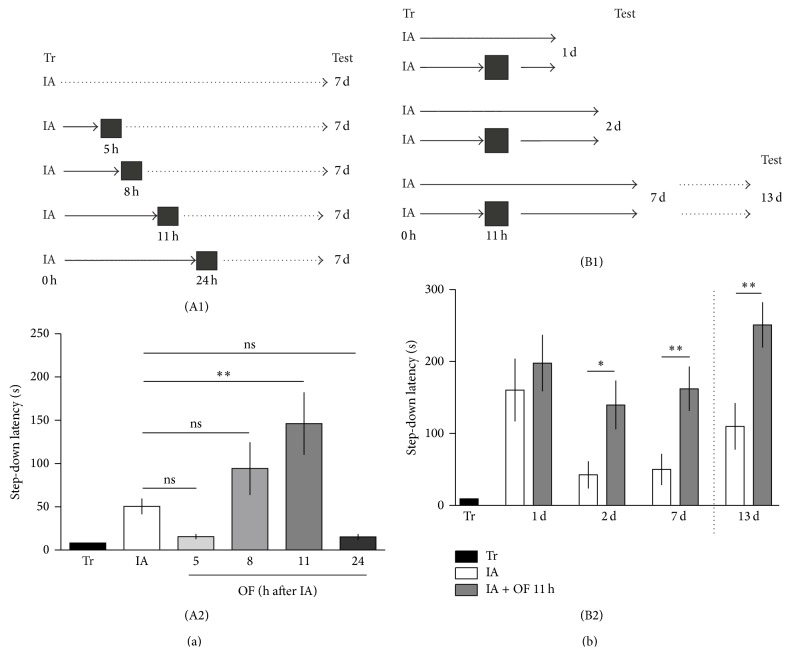
(a) The promoting effect of OF is time-dependent. (A1, B1) Schematic representation of the experimental protocol is presented on the top of each panel. (A2) Animals were trained in the IA and exposed to an OF 5 h, 8 h, 11 h, or 24 h after a weak IA training. Test was performed 7 days after training. Only the exposure to an OF 11 h after training promotes the durability of IA memory. Data are presented as mean ± SEM. (b) Exploration of an open field 11 h after training promotes the persistence of weak IA memory. (B2) Animals were trained in the IA and exposed to an OF 11 h later. Test was performed in independent groups of animals at 1 day, 2 days, and 7 days after training. A retest was performed at 13 days only with the animals that had been tested at 7 days (note a small increase of latency in this control group probably due to the retest effect). Data are presented as mean ± SEM.

After recovery from surgery, animals were handled once a day for two days and then trained in inhibitory avoidance (IA) as described previously [16]. Briefly the apparatus was a 50 × 25 × 25 cm opaque acrylic box whose floor was a grid made of 1 mm caliber stainless steel bars. The left end of the grid was covered by a 12 cm wide, 5.0 cm high platform. During the handling session animals were manipulated in the same way they were during intracerebral infusions. Briefly, they were grasped by hand and slightly restrained in the lap or the arm of the investigator. During the second day of this manipulation in most animals there were no evident signs of stress. For training, animals were gently placed on the platform and, as they stepped down onto the grid, received a single 3 sec, 0.4 mA scrambled foot-shock. The parameter evaluated during training and testing sessions is the latency to step down from the platform. Rats were tested for retention at either 1 day, 2 days, 7 days, or 13 days after training, depending on the experiment. In the test sessions the footshock was omitted and the latency was evaluated for a maximum of 300 seconds. All animals were tested only once (except one group of Figure 1(b)). Training was always performed between 8:30 and 9:30 A.M. For each experiment the number of animals in each group is detailed in the Results.

### 2.4. Drug Infusions

The volume infused was 1 *μ*L/side and the infusion rate was 0.25 *μ*L/min. For intracerebral infusions, 30-Gauge needles connected to Hamilton syringes were used. Infusions were delivered through a needle extending 1 mm beyond the tip of the guide cannula. The needle was left in place for additional 120 sec to minimize backflow. During the procedure, the animals were slightly restrained with the hands, without provoking any evident stress as mentioned in the previous section. Drugs and doses were as follows: SCH 23390, 1.5 *μ*g/side (purchased from Sigma-Aldrich); oligonucleotide pairs (ODNs, Genbiotech, S.R.L) were prepared according to Guzowski et al. [19]. ODNs are chimeric phosphorothioate/phosphodiester, which contained phosphorothioate linkages on the three terminal bases of both the 5′ and 3′ ends and phosphodiester internal bonds.* arc* antisense ODN (*arc* ASO) was directed against a 20 mer sequence (bases 209–228, GenBank accession number U19866) covering the* arc* start site. Missense* arc* ODN (*arc* MSO) containing the same base composition in randomized order served as control. ODNs were dissolved in saline solution and infused into the CA1, in a concentration and volume of 1 nmol/*μ*L per side.

### 2.5. Cannula Placement

To check cannula placement, 24 h after the end of the behavioral procedures, animals were deeply anesthetized and killed by decapitation 15 min later, and histological localization of the infusion sites was established using a binocular magnifying glasses. Coordinates were based on Paxinos and Watson (1997) [18]. Schematic representation of rat brain sections showing the approximated extension of the area (gray) reached by the infusions of 1 *μ*L of methylene blue in the CA1 region of the dorsal hippocampus is shown in Figure 3, which also include a tissue slice showing the position of a cannula. Only data from animals with cannulae located in the intended site were included in the final analysis.

### 2.6. Open Field

The open field was a 50 cm high, 50 cm wide, and 39 cm deep arena with black plywood walls and a brown floor divided into nine squares by black lines. The number of line crossings and rearings was measured manually during each minute, in a 5 min test session. The decrease of these parameters is considered an index of spatial habituation [20].

### 2.7. Data Analysis

Data were analyzed by one-way ANOVA followed by Newman-Keuls multiple comparison, repeated measures, two-way ANOVA followed by Bonferroni comparison test or Student's* t*-test when only two groups were compared. In Figures 1(a) and 1(b) the statistical analysis was performed with Student's* t*-test because each time point represents results of separate experiments. Data in the bar graphs are presented as mean ± SEM.

## 3. Results

To determine whether a maintenance tagging process operates late after training to generate persistent LTM storage we utilized IA training. This task has been extensively used for studying posttraining memory processing because of its rapid hippocampus-dependent acquisition and reliable hippocampus-dependent recall [16, 21]. Moreover, IA training induces LTP in CA1 region [22]. Differences in LTM duration can be achieved by modifying the amount or the strength of IA training. Therefore, we trained rats with a weak protocol in order to induce the expression of a robust short-lasting LTM evaluated at 1 day, but a poor long-lasting LTM tested beyond 2 days after training session. First, rats were exposed to a novel environment 5, 8, 11, or 24 h after training and tested 7 days after training (Figure 1(a)). The enabling effect of OF on long-lasting IA memory was time-dependent, being only effective at 11 h after a weak IA training (Figure 1(a)) (IA versus IA + OF 11 h: ^∗∗^ *P* < 0.01, *n* = 16-17; Student's* t*-test). No effect was seen when rats were exposed to a novel environment 5, 8, or 24 h after training and tested 7 days after training (Figure 1(a)) (IA versus IA + OF 5 h: *P* > 0.05, *n* = 11-12; IA versus IA + OF 8 h: *P* > 0.05, *n* = 10–12, IA versus IA + OF 24 h: *P* > 0.05, *n* = 5; Student's* t*-test). Then, in order to evaluate if OF exposure affects selectively the memory persistence, independent groups of rats were trained in a weak IA, exposed to OF 11 h later and tested at 1, 2, or 7 days after training session (Figure 1(b)). No effects on memory expression were obtained when animals were tested at 1 day after training (1 d, IA versus IA + OF 11 h: *P* > 0.05, *n* = 9-10; Student's* t*-test). As expected, memory expression was significantly increased 2 days after the training session (2 d, IA versus IA + OF 11 h: ^∗^ *P* < 0.05, *n* = 15-16; 7 d, IA versus IA + OF 11 h: ^∗∗^ *P* < 0.01, *n* = 13–15; Student's* t*-test). Even more, 7-day-tested rats that were retested 13 days after training expressed an enhanced long-lasting IA memory (13 d, IA versus IA + OF 11 h: ^∗∗^ *P* < 0.01, *n* = 11-10; Student's* t*-test). These results indicate that spatial novelty specifically enhances long-lasting LTM storage without affecting short-lasting LTM formation. Moreover, the effect of OF on memory persistence occurs late after a weak IA training and in a time-dependent manner, transforming a short-lived IA LTM into a long-lasting IA LTM.

**Figure 2:**
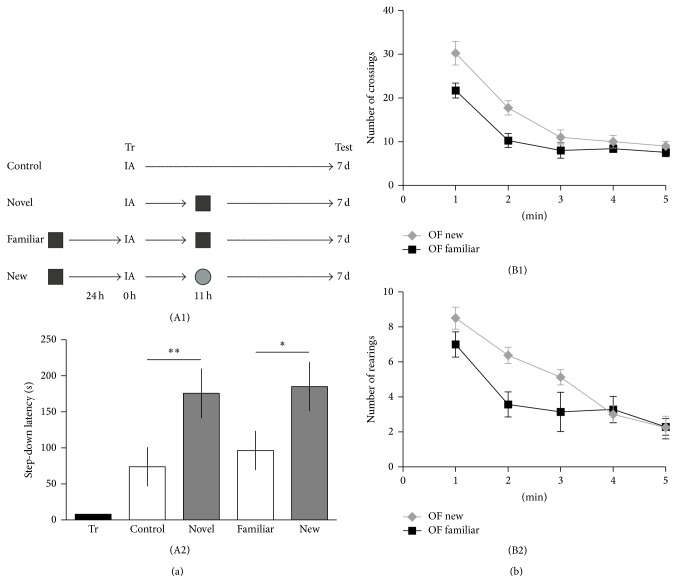
(a) A novel, but not a familiar, open field exposure promotes the durability of IA memory. (A1) Schematic representation of the experimental protocol is presented on the top of the panel. (A2) Animals (“Familiar” and “New” groups) were exposed for 30 min to the OF 24 h before IA training. On the day of the training, we used a different OF only for the labeled “New” group. Novel group of rats were exposed to a single OF session the day of the IA training and the control group of animals did not explore the OF. (b) Bar graph represents the number of quadrant crosses (B1) or rearings (B2) in a new or familiar OF during 5 min. Data are presented as mean ± SEM.

To directly address whether long-lasting LTM is induced by the novel nature of the environment, we subjected animals to an open field 30 min on the previous day. On the day of training, 11 h after a weak IA training, we use the same open field (Familiar group) or a different one with respect to which they were exposed the day before (New group). In contrast to what is observed when a new environment is explored no long-lasting LTM evaluated at 7 days is induced when a familiar environment is presented (Figure 2(a)) (Control versus Novel: ^∗∗^ *P* < 0.05, *n* = 15; Familiar versus New: ^∗^ *P* < 0.05, *n* = 16; Newman-Keuls test after ANOVA). We registered the number of crossings (Figure 2(b), (B1)) and the number of rearings (Figure 2(b), (B2)) during the 5 min new or familiar OF sessions, observing a significant decrease in these parameters in the familiar group of rats (OF New versus OF Familiar; interaction crossings: ^∗^ *P* < 0.05, interaction rearings: ^∗^ *P* < 0.05, repeated measures, two-way ANOVA followed by Bonferroni test). These data reflect the habituated response of rats when they explore a familiar environment. In contrast, a high exploratory activity in the New group is consistent with the recognition of the arena as a novel place.

**Figure 3:**
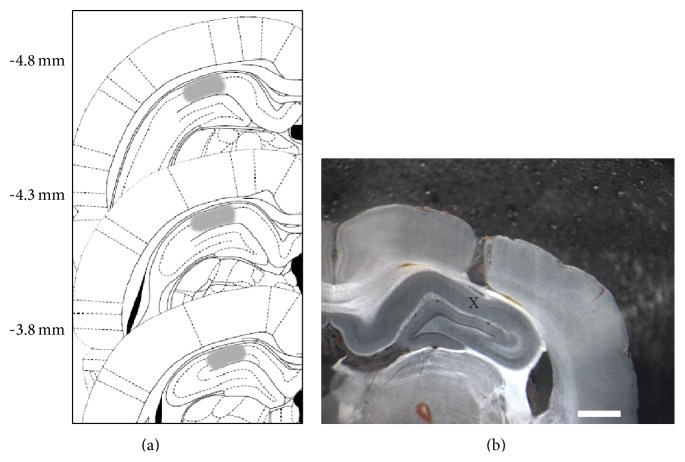
(a) Schematic representation of rat brain sections at three rostrocaudal planes (AP −4.3 mm, LL ± 3.0 mm, DV 1.4 mm from bregma) taken from the atlas of Paxinos and Watson (1997). In stippling, the extension of the area reached by the infusions in the dorsal hippocampus (CA1). (b) Photomicrograph shows the placement of the cannula; the “X” indicates the place corresponding to the area of drug infusion in hippocampus. Scale bar: 1 mm.

**Figure 4:**
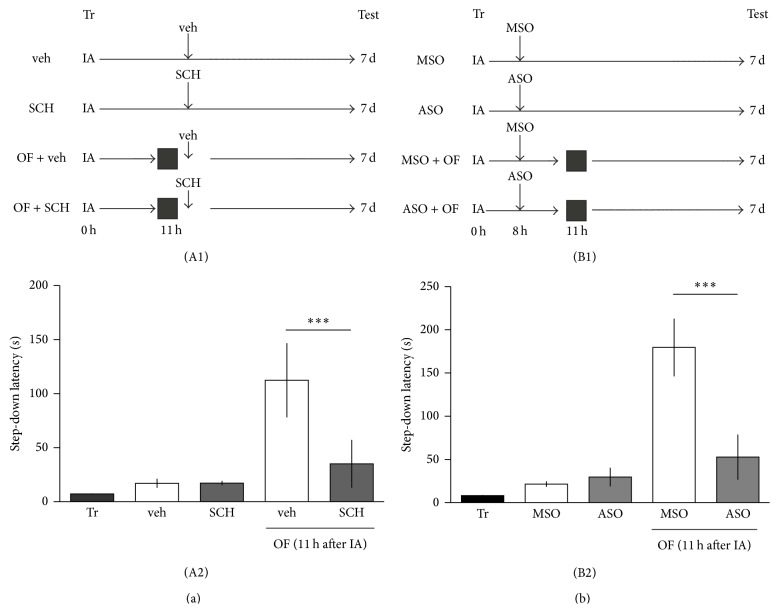
The effect of novelty on the promotion of memory persistence requires dopamine D1/D5 receptors and Arc expression in the dorsal hippocampus. (A1, B1) Schematic representation of the experimental protocol is presented on the top of each panel. (A2) Animals were trained in the IA and exposed to an OF 11 h later. Shortly after exploration, rats were CA1 infused with SCH 23390 (1.5 *μ*g/1 *μ*L per side) or veh. Two other groups of rats were infused with veh or SCH in the absence of OF. Test was performed 7 days after training. (B2) Animals were trained in the IA and 8 h after training they were CA1 infused with antisense (ASO) or missense (MSO) oligonucleotide of* arc*. 3 h later they were exposed to an OF. Two other groups of rats were infused with MSO or ASO in the absence of OF. Test was performed 7 days after training. Data are presented as mean ± SEM.

It is well known that the novelty signal processing involved the release of dopamine in the hippocampus from the ventral tegmental area (VTA) [23]. Moreover, novel exploration was suggested to induce D1/D5 protein synthesis-dependent process in the hippocampus [7]. Dopaminergic neurons of the VTA innervate the CA1 region in the hippocampus [24] and these dopaminergic connections also control the late posttraining protein synthesis- and BDNF-dependent persistence of LTM storage via activation of D1/D5 receptor [16, 21]. To study if the promoting effect of novel OF on long-lasting IA LTM was dependent on hippocampal D1/D5 functionality, rats were CA1-infused (Figure 3) with SCH 23390 (1.5 *μ*g/1 *μ*L per side), an antagonist of D1/D5 dopamine receptors, shortly after OF exploration at 11 h after IA training. As shown in Figure 4(a), SCH 23390 blocked IA LTM expression at 7 days (veh versus SCH: *P* > 0.05, *n* = 12-13; OF (11 h after IA) veh versus SCH: ^∗∗∗^ *P* < 0.001, *n* = 12-13; Newman-Keuls test after ANOVA), indicating that hippocampal D1/D5 receptors are required for novelty-induced promotion of LTM persistence.

Which are the PRPs important for the maintenance of LTM storage? We and others demonstrated that a late posttraining increase in the expression of BDNF is essential for the persistence of LTM storage [16, 17, 25]. Arc is a well-known PRP whose expression is controlled by BDNF [26], and the exposure to a novel OF increased hippocampal Arc levels [27, 28]. We recently demonstrated that the local infusion of* arc* antisense oligonucleotides (ASO) 3 h before a novel OF session impaired the increase in Arc protein levels observed 30 min after OF exposure [28]. Therefore, we next determined whether Arc expression in the dorsal hippocampus is required for novelty-induced promotion of long-lasting IA memory. As shown in Figure 4(b), intrahippocampal CA1 infusion of* arc* ASO 8 h after IA training session prevented the spatial novelty-induced promotion of long-lasting IA memory observed in control group of rats injected with an* arc* missense oligonucleotide (MSO) (OF (11 h after IA), MSO versus ASO: ^∗∗∗^ *P* < 0.001, *n* = 14-15; Newman-Keuls test after ANOVA) when tested 7 days after IA training.

## 4. Discussion

The main finding of the present study is that a consolidated but nonpersistent memory presents a delayed and transient time window 11 h after the learning session in which it would be possible to use proteins/products derived from a separate novel experience, in order to make it persistent. This fact led us to postulate the idea that a maintenance tagging process is essentially involved in establishing a persistent IA LTM. This could recapitulate the setting of a learning tag, at the moment of memory encoding, which is required to form LTM through a behavioral tagging process [3, 29].

Tonegawa and colleagues [30] first employed the term maintenance tag to explain results consistent with the existence of a mark that capture protein components required for stabilization of synaptic plasticity at three days, but not at one day, after induction in* Aplysia* [31]. Here we describe behavioral and pharmacological experiments supporting the idea that a maintenance tagging process operates late after training session in order to establish a persistent IA LTM. This is based on the following findings: (1) A long-lasting LTM is established when a weak IA training is associated with the exploration of a novel environment in a critical time window around 11 h late after training; novelty experienced outside of this time point is not effective. At 11 h after training the system is prepared (tagged) to use the products derived from the novelty experience (Figure 1(a)). (2) The exploration of a novel environment prevents the decay of LTM at 2 or 7 days; namely, it promotes the persistence of IA-LTM (Figure 1(b)). (3) The exploration of a familiar environment does not induce a persistent LTM (Figure 2). (4) The promoting action of spatial novelty on the persistence of LTM over 7 days depends on the activation of D1/D5 dopamine receptors in the dorsal hippocampus at the moment of novelty exploration (Figure 4(a)). (5) Finally, we demonstrate that the OF effect depends on the induction of Arc expression in the dorsal hippocampus, showing the requirement of Arc protein to ensure the durability of IA-LTM (Figure 4(b)). Thus, these findings show that the persistence of LTM could be determined by behavioral events experienced by subjects long time away the encoding of the information, by using PRPs provided by those events. These findings are in accordance with those reporting that a persistent IA LTM is obtained by infusion of SKF 38393, an agonist of D1/D5 receptors, into the dorsal CA1 of weak IA-trained rats [21] during a restricted time window comparable to that of the novelty exploration-induced IA LTM persistence. Moreover, it was found that BDNF expression in the hippocampus, controlled by D1/D5 receptors, is required late after training for the persistence of LTM storage [16]; and that BDNF infused in the dorsal hippocampus is sufficient to induce long-lasting LTM in animals trained with a weak IA protocol [17]. It was previously shown that hippocampal administration of SCH 12 h after a strong IA training impaired the persistence of IA-LTM (tested 7 days after training). However, this effect was overcome by the local administration of BDNF [21]. These experiments suggest that SCH did not affect the setting or the establishment of the “maintenance tag,” because the administration of one PRP (BDNF) could recover the IA-LTM. The most parsimonious explanation is that D1/D5 activation triggers protein synthesis required for LTM to persist. Together, findings fit well with the idea that a weak training creates a transient maintenance mark or tag late after training that captures PRPs (like Arc) induced by novelty exploration. Our present results together with published data [16, 17, 21, 32] suggest that novel, but not familiar, OF induces D1/D5 receptor activation and Arc expression in the dorsal hippocampus probably due to BDNF action on TrkB receptors [26]. Arc represents a key candidate to be a PRP because its mRNA accumulates* in vivo* near activated synapses in the hippocampus, and it is locally translated [33]. Moreover, the involvement of Arc in the promotion of IA LTM formation was demonstrated recently [28].

It has been recently reported that other interventions than a novel OF during the late consolidation phase of a training inducing a short-lasting LTM promoted the establishment of long-lasting LTM. The stress or the administration of corticosterone 12 h after a contextual fear conditioning selectively prolongs the persistence of this LTM [34]. These effects were prevented by systemic administration of metyrapone, a corticosterone synthesis inhibitor. As glucocorticoid receptors have transcriptional effects on some target genes in hippocampus [35, 36], we suggest that stress and corticosterone probably act providing PRPs required for maintenance tagging process to induce a long-lasting LTM.

In this context, a neuromodulatory effect should be considered. Neuromodulation is a physiological process which alters cellular and synaptic properties via widespread projections [37]. It is generated by the release of a neurotransmitter, induced by an event or by the use of a drug. The well-known modulatory effects on the strength of memory could result from regulation of protein synthesis, which also are essential for the formation and persistence of LTM [7, 21]. However, the main feature of the behavioral tagging hypothesis involved the postulation of a specific transient tag set by the learning experience to be remembered, which captured/utilized PRP. We think that neuromodulation could help provide the PRPs, but they will only be useful in a restricted time window delimited by the tag's kinetics.

Considering STC hypothesis and its behavioral tagging translation, we propose that learning experience could signal two separate marks, where PRPs will be captured in order to allow memory consolidation and persistence. The most parsimonious molecular mechanism to explain these phenomena is that the tags induced by the original training are active at two different moments: first, during encoding for LTM formation and, second, many hours later allowing the establishment of long-lasting memory storage. We also reasoned that a significant proportion of synapses that are tagged by the original experience and that capture PRPs required for the formation of LTM are also marked many hours later and capture PRPs required for the durability of the memory trace. The current vision of STC hypothesis considers the tag as an ensemble of molecules tending to modify the morphology of the dendrite [2, 38]: candidates for synaptic tags during long-term potentiation or during encoding of learning tasks include two protein kinases activated by NMDA receptors, CaMKII and PKA [7, 39], and the BDNF receptor TrkB [6]. However, the molecular underpinnings and the dynamics of the proposed maintenance tag deserve further examination.

An important behavioral implication of our findings is that the durability of memory depends not only on events occurring at the moment of their encoding, but also on other events occurring late after learning. The idea that a maintenance tagging process participates in memory duration provides a novel behavioral approach and a wide framework to explain reinforcements and impairments in memory durability due to interventions occurring during the late consolidation phase of long-lasting memories.

## References

[1] Frey U., Morris R. G. M. (1997). Synaptic tagging and long-term potentiation. *Nature*.

[2] Redondo R. L., Morris R. G. M. (2011). Making memories last: the synaptic tagging and capture hypothesis. *Nature Reviews Neuroscience*.

[3] Moncada D., Viola H. (2007). Induction of long-term memory by exposure to novelty requires protein synthesis: evidence for a behavioral tagging. *Journal of Neuroscience*.

[4] Ballarini F., Moncada D., Martinez M. C., Alen N., Viola H. (2009). Behavioral tagging is a general mechanism of long-term memory formation. *Proceedings of the National Academy of Sciences of the United States of America*.

[5] Wang S.-H., Redondo R. L., Morris R. G. M. (2010). Relevance of synaptic tagging and capture to the persistence of long-term potentiation and everyday spatial memory. *Proceedings of the National Academy of Sciences of the United States of America*.

[6] Lu Y., Ji Y., Ganesan S. (2011). TrkB as a potential synaptic and behavioral tag. *The Journal of Neuroscience*.

[7] Moncada D., Ballarini F., Martinez M. C., Frey J. U., Viola H. (2011). Identification of transmitter systems and learning tag molecules involved in behavioral tagging during memory formation. *Proceedings of the National Academy of Sciences of the United States of America*.

[8] Almaguer-Melian W., Bergado-Rosado J., Pavón-Fuentes N., Alberti-Amador E., Mercerón-Martínez D., Frey J. U. (2012). Novelty exposure overcomes foot shock-induced spatial-memory impairment by processes of synaptic-tagging in rats. *Proceedings of the National Academy of Sciences of the United States of America*.

[9] Cassini L. F., Sierra R. O., Haubrich J. (2013). Memory reconsolidation allows the consolidation of a concomitant weak learning through a synaptic tagging and capture mechanism. *Hippocampus*.

[10] Salvetti B., Morris R. G. M., Wang S.-H. (2014). The role of rewarding and novel events in facilitating memory persistence in a separate spatial memory task. *Learning and Memory*.

[11] Myskiw J. D. C., Furini C. R. G., Benetti F., Izquierdo I. (2014). Hippocampal molecular mechanisms involved in the enhancement of fear extinction caused by exposure to novelty. *Proceedings of the National Academy of Sciences of the United States of America*.

[12] Viola H., Ballarini F., Martínez M. C., Moncada D. (2014). The tagging and capture hypothesis from synapse to memory. *Progress in Molecular Biology and Translational Science*.

[13] Dong Z., Gong B., Li H. (2012). Mechanisms of hippocampal long-term depression are required for memory enhancement by novelty exploration. *Journal of Neuroscience*.

[14] de Carvalho Myskiw J., Benetti F., Izquierdo I. (2013). Behavioral tagging of extinction learning. *Proceedings of the National Academy of Sciences of the United States of America*.

[15] Ballarini F., Martínez M. C., Díaz Perez M., Moncada D., Viola H. (2013). Memory in elementary school children is improved by an unrelated novel experience. *PLoS ONE*.

[16] Bekinschtein P., Cammarota M., Igaz L. M., Bevilaqua L. R. M., Izquierdo I., Medina J. H. (2007). Persistence of long-term memory storage requires a late protein synthesis- and BDNF- dependent phase in the hippocampus. *Neuron*.

[17] Bekinschtein P., Cammarota M., Katche C. (2008). BDNF is essential to promote persistence of long-term memory storage. *Proceedings of the National Academy of Sciences of the United States of America*.

[18] Paxinos G., Watson C. (1997). *The Rat Brain in Stereotaxic Coordinates*.

[19] Guzowski J. F., Lyford G. L., Stevenson G. D. (2000). Inhibition of activity-dependent arc protein expression in the rat hippocampus impairs the maintenance of long-term potentiation and the consolidation of long-term memory. *The Journal of Neuroscience*.

[20] Winograd M., Viola H. (2004). Detection of novelty, but not memory of spatial habituation, is associated with an increase in phosphorylated cAMP response element-binding protein levels in the hippocampus. *Hippocampus*.

[21] Rossato J. I., Bevilaqua L. R. M., Izquierdo I., Medina J. H., Cammarota M. (2009). Dopamine controls persistence of long-term memory storage. *Science*.

[22] Whitlock J. R., Heynen A. J., Shuler M. G., Bear M. F. (2006). Learning induces long-term potentiation in the hippocampus. *Science*.

[23] Lisman J. E., Grace A. A. (2005). The hippocampal-VTA loop: controlling the entry of information into long-term memory. *Neuron*.

[24] Gasbarri A., Verney C., Innocenzi R., Campana E., Pacitti C. (1994). Mesolimbic dopaminergic neurons innervating the hippocampal formation in the rat: a combined retrograde tracing and immunohistochemical study. *Brain Research*.

[25] Ou L.-C., Yeh S.-H., Gean P.-W. (2010). Late expression of brain-derived neurotrophic factor in the amygdala is required for persistence of fear memory. *Neurobiology of Learning and Memory*.

[26] Schratt G. M., Nigh E. A., Chen W. G., Hu L., Greenberg M. E. (2004). BDNF regulates the translation of a select group of mRNAs by a mammalian target of rapamycin-phosphatidylinositol 3-kinase-dependent pathway during neuronal development. *Journal of Neuroscience*.

[27] Vazdarjanova A., Ramirez-Amaya V., Insel N. (2006). Spatial exploration induces ARC, a plasticity-related immediate-early gene, only in calcium/calmodulin-dependent protein kinase II-positive principal excitatory and inhibitory neurons of the rat forebrain. *Journal of Comparative Neurology*.

[28] Martínez M. C., Alen N., Ballarini F., Moncada D., Viola H. (2012). Memory traces compete under regimes of limited Arc protein synthesis: implications for memory interference. *Neurobiology of Learning and Memory*.

[29] Bethus I., Tse D., Morris R. G. M. (2010). Dopamine and memory: modulation of the persistence of memory for novel hippocampal NMDA receptor-dependent paired associates. *Journal of Neuroscience*.

[30] Kelleher R. J., Govindarajan A., Tonegawa S. (2004). Translational regulatory mechanisms in persistent forms of synaptic plasticity. *Neuron*.

[31] Casadio A., Martin K. C., Giustetto M. (1999). A transient, neuron-wide form of CREB-mediated long-term facilitation can be stabilized at specific synapses by local protein synthesis. *Cell*.

[32] Katche C., Dorman G., Gonzalez C. (2013). On the role of retrosplenial cortex in long-lasting memory storage. *Hippocampus*.

[33] Farris S., Lewandowski G., Cox C. D., Steward O. (2014). Selective localization of Arc mRNA in dendrites involves activity- and translation-dependent mRNA degradation. *Journal of Neuroscience*.

[34] Yang C., Liu J.-F., Chai B.-S. (2013). Stress within a restricted time window selectively affects the persistence of long-term memory. *PLoS ONE*.

[35] Datson N. A., Van der Perk J., De Kloet E. R., Vreugdenhil E. (2002). Identification of corticosteroid-responsive genes in rat hippocampus using serial analysis of gene expression. *European Journal of Neuroscience*.

[36] Roozendaal B., Hernandez A., Cabrera S. M. (2010). Membrane-associated glucocorticoid activity is necessary for modulation of long-term memory via chromatin modification. *The Journal of Neuroscience*.

[37] Kupfermann I., Cohen J. L., Mandelbaum D. E., Schonberg M., Susswein A. J., Weiss K. R. (1979). Functional role of serotonergic neuromodulation in Aplysia. *Federation Proceedings*.

[38] Ramachandran B., Frey J. U. (2009). Interfering with the actin network and its effect on long-term potentiation and synaptic tagging in hippocampal CA1 neurons in slices in vitro. *The Journal of Neuroscience*.

[39] Redondo R. L., Okuno H., Spooner P. A., Frenguelli B. G., Bito H., Morris R. G. M. (2010). Synaptic tagging and capture: differential role of distinct calcium/calmodulin kinases in protein synthesis-dependent long-term potentiation. *Journal of Neuroscience*.

